# Effect of nutritional interventions on the psychological symptoms of premenstrual syndrome in women of reproductive age: a systematic review of randomized controlled trials

**DOI:** 10.1093/nutrit/nuae043

**Published:** 2024-04-29

**Authors:** Jazz Robinson, Amy Ferreira, Marina Iacovou, Nicole J Kellow

**Affiliations:** Department of Nutrition, Dietetics and Food, Monash University, Notting Hill, Victoria, Australia; Department of Nutrition, Dietetics and Food, Monash University, Notting Hill, Victoria, Australia; Department of Molecular and Translational Science, Centre of Innate Immunity and Infectious Diseases, Hudson Institute of Medical Research, Clayton, Victoria, Australia; Department of Nutrition, Dietetics and Food, Monash University, Notting Hill, Victoria, Australia; Department of Molecular and Translational Science, Centre of Innate Immunity and Infectious Diseases, Hudson Institute of Medical Research, Clayton, Victoria, Australia

**Keywords:** diet, nutritional supplements, premenstrual dysphoric disorder, premenstrual syndrome, psychological health

## Abstract

**Context:**

Premenstrual syndrome (PMS) affects approximately 48% of women of reproductive age worldwide. It can lead to functional impairment, lower quality of life, and decreased work productivity. Despite the availability of medical treatment options, women are seeking alternative interventions because of concerns of harmful side effects and limited evidence of efficacy associated with pharmacological treatments. To date, high-quality research investigating the effects of dietary and nutrient intervention on PMS is limited.

**Objective:**

This systematic review investigated the effect of nutritional interventions on the psychological symptoms of PMS.

**Data Sources:**

Five electronic databases were searched for randomized controlled trials (RCTs) published in English from inception to October 2022. Trials eligible for inclusion were nutritional intervention studies involving women of reproductive age that measured PMS-associated psychological outcomes.

**Data Extraction:**

Articles were selected using prespecified inclusion criteria. Data screening and extraction and risk-of-bias assessments were conducted by 3 independent reviewers using article screening software and the Cochrane Risk of Bias 2 tool.

**Data Analysis:**

Thirty-two articles reporting on 31 RCTs involving 3254 participants, ranging in age from 15 to 50 years were included and narratively reviewed. Only 1 of the included studies had a low risk of bias. Treatment with vitamin B_6_, calcium, and zinc consistently had significant positive effects on the psychological symptoms of PMS. There was insufficient evidence to support the effects of vitamin B_1_, vitamin D, whole-grain carbohydrates, soy isoflavones, dietary fatty acids, magnesium, multivitamin supplementation, or PMS-specific diets.

**Conclusions:**

There is some evidence to support the use of nutritional interventions for improving psychological symptoms of PMS. However, more research using consistent protocols, procedures to minimize risk of bias, intention-to-treat analysis, and clearer reporting is required to provide conclusive nutritional recommendations for improving PMS-related psychological outcomes.

**PROSPERO registration no:**

CRD42022369999.

## INTRODUCTION

Millions of reproductive-age women worldwide are affected by adverse premenstrual symptoms ranging from mild to severe.^1^ It is estimated that 48% of women experience premenstrual syndrome (PMS), and 3%–8% suffer from premenstrual dysphoric disorder (PMDD), a more severe and debilitating form of PMS.^2^ Despite the high prevalence of PMS/PMDD, many healthcare professionals and the general community are unaware of the profound impact of symptoms on an individual’s social, interpersonal, and working life.^1^^,^^3^ PMS/PMDD can present at around 14–15 years of age and persist until menopause (average age, 51 years), with symptoms recurring monthly for approximately 480 susceptible cycles, potentially leading to increased functional impairment, lower quality of life, reduced work and school productivity, and indirect economic costs, imposing a lifelong burden on affected individuals.^1^^,^^4^^,^^5^ Despite this, the impact of menstrual-associated symptoms is frequently underestimated and underreported, the pathophysiology remains elusive, and it remains an area of women’s health that is severely under-researched.^3^

PMS is defined as “at least one emotional, physical or behavioral symptom that increases in severity during the luteal phase of the menstrual cycle (1–2 weeks before menstruation), and resolves by the start or within a few days of menstruation.”^6^ Symptoms must interfere with social, work, or school performance and be present during 2 menstrual cycles of prospective recording.^2^ The American Psychiatry Association has published strict diagnostic criteria for PMDD in the *Diagnostic and Statistical Manual of Mental Disorders* (Fifth Edition) (*DSM-V*).^7^ Diagnosis of PMDD requires the presence of at least 5 affective or somatic premenstrual symptoms, including at least 1 mood symptom.^8–10^ There are more than 200 recorded symptoms for PMS/PMDD, with the most common often categorized into physical symptoms (fatigue, weight gain, abdominal bloating, headache, acne, breast tenderness, and food craving), and emotional and behavioral symptoms (crying, anxiety, depression, irritability, confusion, low mood, and anger).^5^

The exact mechanisms involved in the pathophysiology of PMS-associated emotional symptoms are currently unknown; however, cyclical fluctuations in estrogen and progesterone, key hormones central to the regulation of the female reproductive system and menstrual cycle, are thought to play a large role. Estradiol, an estrogen steroid hormone, modulates serotonin levels by altering the expression of 5-HT 2A receptors and serotonin transporter genes in brain regions associated with behavior and emotion.^11–13^ Women with PMS/PMDD have serotonin deficits during the luteal phase of the menstrual cycle when estradiol levels decline,^14^^,^^15^ suggesting a possible increased sensitivity to the effects of estradiol on serotonin regulation. Variations in progesterone and allopregnanolone, the main metabolite of progesterone, have also been implicated in PMS/PMDD.^1^ Preclinical research indicates that declining progesterone levels during the late luteal phase are associated with increased anxiety and alterations in γ-aminobutyric acid (GABA) receptor function.^16^^,^^17^ Under normal conditions, allopregnanolone positively modulates the GABA(A) receptor, resulting in sedative effects.^18^ In women with PMS/PMDD, hypotheses propose decreased GABA(A) receptor sensitivity to neuroactive steroids, like progesterone, and/or lower allopregnanolone levels may result in depressive and anxiety symptoms.^19^ In addition to hormonal alterations, emerging research implicates brain-derived neurotrophic factor (BDNF) in PMS/PMDD pathogenesis, although its exact role has yet to be elucidated. Although BDNF levels fluctuate throughout the menstrual cycle,^20^ serum BDNF levels are significantly higher in women with PMDD than in women without PMDD during the luteal phase.^21^ Conversely, women with PMS exhibit the reverse pattern.^22^

Current management of PMS/PMDD includes pharmacological agents (antidepressant and hormonal medications), or surgery (removal of ovaries, removal of ovaries and fallopian tubes, or total hysterectomy).^3^^,^^23^ Selective serotonin reuptake inhibitors, a class of antidepressants, can alleviate emotional and behavioral PMS symptoms through maintenance of serotonin levels with strong efficacy.^23^ However, adverse side effects are possible (eg, insomnia, nausea, sexual dysfunction) and PMS symptoms recur upon drug discontinuation.^3^^,^^23^ Hormonal treatments such as oral contraceptives, estrogen therapy, and gonadotropin-releasing hormone analogs (to induce temporary menopause) are also available.^23^ In recent years, more women are seeking drug-free alternatives for PMS/PMDD management due to concerns regarding side effects and variable evidence of efficacy for pharmacological treatments.

Many nonpharmacological approaches to PMS management involve positive health and lifestyle practices, including exercise, cognitive behavioral therapy, and dietary changes, although evidence from robust research is limited and conflicting.^24^^,^^25^ Despite this, several recent studies investigating the effects of nutritional interventions on PMS show promising results. Kwon et al^26^ determined that high amounts of bread and snack consumption and low adherence to a Mediterranean diet (characterized by high consumption of whole grains, nuts, olive oil, legumes, fruits, and vegetables) were associated with greater risk of PMS.^27^^,^^28^ The Mediterranean diet’s abundance in vitamins, antioxidants, polyphenols, and unsaturated fats may reduce symptoms of PMS by attenuating oxidative stress.^27^ Additionally, several studies report that a Western dietary pattern, characterized by high intake of energy-dense foods containing large amounts of sugar, saturated fat, and salt, is associated with inflammatory biomarkers (eg, cytokines) and a higher prevalence and severity of PMS symptoms.^28^^,^^29^ The limitations of these studies include cross-sectional design, which prevents the establishment of a causal link between PMS and nutrition interventions; measurement of dietary intake through food frequency questionnaires, which are often subject to participant recall bias; and limited generalizability.^26–29^

Bertone-Johnson et al^30^ suggested that high intakes of foods containing calcium and vitamin D may reduce risk of PMS development, because circulating levels fluctuate across the menstrual cycle in response to endogenous estrogen changes. Zinc may also alleviate PMS symptoms through its antioxidant and anti-inflammatory properties.^31^ Water-soluble B-group vitamins are involved in the metabolism of neurotransmitters.^32^ Among them, vitamin B_6_ acts as a cofactor in the synthesis of serotonin from tryptophan and may reduce PMS symptoms, similar to the effects of broad-spectrum multivitamins.^32^

Overall, current research to support the effectiveness of dietary interventions on PMS is limited due to weak study designs, inconsistencies in the assessment of psychological PMS symptoms, and inadequate reporting of study methods. In this systematic review, we aimed to synthesize the outcomes of published randomized controlled trials (RCTs) exploring the effects of nutrition-based interventions on psychological symptoms of premenstrual syndrome in women of reproductive age.

## METHODS

This review was conducted in accordance with the Preferred Reporting Items for Systematic Reviews and Meta-Analyses (PRISMA) Statement.^33^ The protocol of this systematic review was registered in the PROSPERO database in November 2022 (registration no. CRD42022369999).

### Search strategy and study selection

Table 1 outlines the PICOS framework used to define the research question and the study inclusion and exclusion criteria. We searched for published studies via the following electronic databases from inception to October 2022: Ovid Medline, Scopus, CINAHL Plus, Embase, and the Cochrane Database of Systematic Reviews. The search strategy is depicted in Table S1 in the Supporting Information online. Non-nutritive interventions such as herbal extracts or spices were excluded from the search because they have recently been reviewed elsewhere.^24^ Search results were integrated into a systematic review screening software program (Covidence Systematic Review Software, Veritas Health Innovation, Melbourne, VIC, Australia), where duplicates were automatically removed, and remaining articles were screened for eligibility. Article screening was conducted independently by 2 review authors (A.F. and J.R.) and resolved by a third reviewer (N.J.K. or M.I.).

**Table 1:** PICOS criteria for inclusion of studies

	Inclusion criterion	Exclusion criterion
Population	Menstruating women of reproductive age and/or women of reproductive age with a diagnosis of premenstrual syndrome or premenstrual dysphoric disorder	Studies involving nonhumans; men; children; nonmenstruating or postmenopausal women; pregnant women; women using hormone replacement; women with endometriosis, polycystic ovary syndrome, adenomyosis, hysterectomy, thyroid disorders, endocrine disorders, irritable bowel disease, cancers (other than nonmelanoma skin cancers)
Intervention	Provision of foods, nutrients, or nutritional advice as part of a clinical trial intervention; assessment of dietary intake via prospective collection of dietary intake data using validated food frequency questionnaires, food records, or via retrospective dietary recall methods	Provision of foods, nutrients, or /dietary advice in conjunction with other interventions (eg, physical activity, yoga, pharmacological treatments, psychological counselling); provision of herbal supplements or spices; assessment of dietary intake using nonvalidated methods; studies that only measure blood markers of nutritional status
Comparison	Control group with or without premenstrual syndrome or premenstrual dysphoric disorder diagnosis	No control group or control intervention
Outcome	Measurement of premenstrual syndrome or premenstrual dysphoric disorder symptoms (total score) and/or menstruation-associated psychological symptoms using validated tools	Measurements made without the use of any validated tools or official diagnostic criteria or measurements of only nonpsychological symptoms
Study type	Randomized controlled trials	Cohort, cross sectional, case-control, and qualitative studies

### Data extraction and risk-of-bias assessment

Data were extracted independently by 2 review authors (A.F. and J.R.) and verified by a third reviewer (N.J.K.), and included the following: author, date of publication, country of publication, study design (including blinding, if applicable), participant characteristics, method of PMS/PMDD assessment, intervention details (eg, type, quantity of foods or supplements, duration, comparator used, method to assess compliance), tools used to assess psychological outcomes, and funding source. Outcomes were reported as the effect of intervention on psychological outcomes, in comparison to placebo or control groups.

Risk of bias for included studies was independently assessed by 2 reviewers (A.F. and J.R.) using the Cochrane Risk of Bias Tool 2.0 (RoB2)^34^ for assessment of RCTs or for crossover trials, where applicable. This tool is used to assess study risk of bias by identifying potential sources of selection bias (random sequence generation and allocation concealment), performance bias (blinding of participants and personnel), detection bias (blinding of outcome assessment), attrition bias (incomplete outcome data), and reporting bias (selective reporting). Individual domains were assessed for adherence to bias minimization items, and overall bias for each study was classified as “low” risk of bias, “some concerns,” or “high” risk of bias. Any discrepancies in the authors’ risk-of-bias assessments were resolved by consensus and discussion with a third reviewer (N.J.K.).

## RESULTS

### Search results

**Figure 1:**
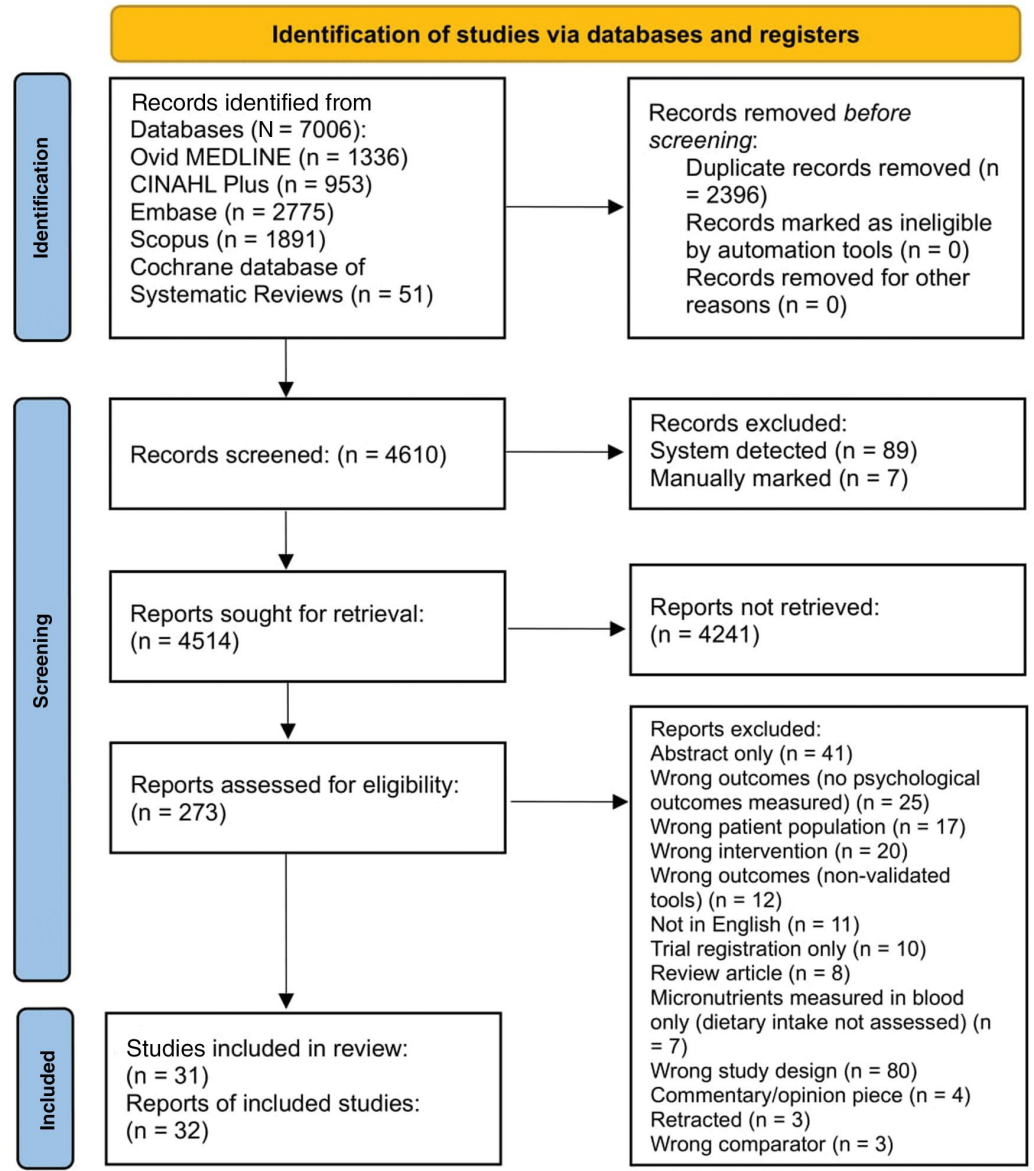
PRISMA flow diagram of included studies and reasons for exclusion at each screening stage.

A PRISMA flow diagram of the article selection process is presented in Figure 1. Initial database searches identified a total of 7006 articles. After duplicates were removed (n = 2396) and nonrelevant articles excluded (n = 4241), 273 papers were subjected to full-text review. From these, 32 articles (reporting on 31 trials) met the inclusion criteria and were available for qualitative synthesis.^35–66^

### Study characteristics

Characteristics of the included studies are detailed in Table 2.^35–66^ Articles were published from 1985 to 2022 and studies were conducted worldwide: Iran (n = 11), the United States (n = 8), United Kingdom (n = 5), Italy (n = 2), and Turkey (n = 2). Of the remaining trials, 1 was conducted in each of the following countries: Norway, New Zealand, and Brazil. A total of 3254 participants, ranging in age from 15 to 50 years, were involved in the published trials. The sample sizes of individual studies varied, with the number of total participants ranging from 14 to 497. In the included studies, a wide variety of nutritional interventions were assessed; therefore, results were summarized within 6 main categories: vitamins, minerals, multivitamins, dietary fat, carbohydrate, and other interventions. Because of the heterogeneity in the mean age of participants (18–37 years), nutritional interventions provided, duration of treatment (1–6 months), and outcome measurement techniques used in studies included in this review, a meta-analysis of study results was not possible. Therefore, this review focuses on a narrative synthesis of study outcomes.

**Table 2:** Characteristics of included studies

Reference	Country of origin; study design	Sample size, participant characteristics	Method of PMS assessment	Intervention and comparator (placebo)	Method used to assess compliance to intervention and study completion rate	Outcome/s measured; tools used to assess outcomes	Effect of intervention compared with placebo on psychological outcomes
Abdollahifard et al (2014)^35^	Iran; parallel RCT, double blinded	n = 100 female university students with PMS randomized to either intervention (n = 50) or control (n = 50) groups. Mean age ± SD: 21.2 ± 0.8 y (intervention group), 21.4 ± 0.54 y (control group)	A daily status record form containing 20 symptoms of PMS, which were adopted from *DSM-IV* criteria, was used. PMS symptoms included: tension, ambivalence, irritability, anxiety, depression, forgetfulness, suicidal ideation, poor concentration, and crying. Participants marked the severity of their daily symptoms on a Likert scale.	Intervention: 2 × 100 mg of vitamin B_1_ pills/d Placebo: 2 × 100 mg starch pills/d Length of intervention: 2 mo	Participants were sent a text message every week and attended the university every 2 wk for a review. n = 80 of 100 participants completed the study	Daily status record form (content validity assessment of the form was undertaken by the authors)	↓ Severity of combined mental symptoms
Abdollahi et al (2019)^36^	Iran; parallel RCT, double blinded	n = 146 female university students with PMS and deficient in vitamin D, randomized into intervention (n = 73) or control (n = 73) groups Mean age ± SD: 22.5 ± 2.6 y (intervention group), 22.7 ± 3.0 y (placebo group). All participants had a serum 25-(OH)-D < 20 ng/mL	Participants were assessed as having PMS if they had >1 somatic or mood symptom for 5 d before menstruation, in 3 previous cycles, that disappeared at the onset of menstruation. Mood symptoms included depression, jitteriness, anxiety, dizziness, low concentration, and impaired social activity.	Intervention: 1 × 2000 IU vitamin D pill every second day Placebo: 1 × maltodextrin pill every second day Length of intervention: 12 wk	Assessed by pill counts at the end of the each 4-wk period n = 130 of 146 participants completed the study	PSST questionnaire (Iranian version) consists of 19 items in 2 sections. The first section contains 14 items of PMS symptoms, and the second section contains 5 items evaluating the effects of symptoms on women with PMS.	↔ Severity of total PMS symptoms
Behboudi-Gandevani et al (2018)^37^	Iran; parallel RCT	n = 95 women with PMS and BMI 18.5–25 kg/m^2^ randomized into intervention (n = 47) or control (n = 48) groups. Mean ± SD age: 24.22 ± 2.9 y (intervention group), 23.64 ± 3 y (control group).	Not stated. Participants were referred to study via general practitioners.	Intervention: 2 × pills/d containing 1 g fish oil with 180 mg eicosapentaenoic acid and 120 mg docosahexaenoic acid Placebo: 2 × placebo pills/d (free of oil) (composition not stated) Length of intervention: 10 consecutive days per month (8 d before and 2 d after commencement of menses), for 3 mo	Not stated. n = 90 of 95 participants completed study	PSST questionnaire (Iranian version); SF-12 survey used to assess quality of life	↓ Severity of PMS symptoms (excluding overeating and insomnia) ↔ quality of life
Bryant et al (2005)^38^	United Kingdom; cross-over RCT, double blinded	n = 41 women with PMS and BMI 19–30 kg/m^2^. Mean ± SD age: 33.65 ± 5.85 y.	Using the DSR, 17 symptoms were rated on a 5-point Likert scale. Participants were required to demonstrate an increase in overall symptoms that was > 30% greater premenstrually (6 d before menstruation) than during days 5–10 after onset of menses for ≥1 of 2 screening mo.	Intervention: 1 serving/d of supplement powder + snack bar containing total of 68 mg/d soy isoflavones Placebo: 1 serving/d of supplement powder + snack bar containing milk protein. Length of intervention: 2 menstrual cycles	4 × 24 h urine samples to measure isoflavone metabolism during the premenstrual phase of each intervention treatment cycle. n = 23 of 41 participants completed the study	DSR	↔ Severity of total PMS symptoms
Dadkhah et al (2016)^39^	Iran; parallel RCT, double blinded	n = 86 women with PMS randomized into intervention (vitamin E; n = 28; vitamin D; n = 30), or control (n = 28) groups. Mean age ± SD: 30.9 ± 7.0 y (vitamin E), 30.82 ± 6.1 y (vitamin D), 29.64 ± 5.5 y (control group).	Participants with PMS according to the *DSM-IV* criteria of the American Psychiatry Association completed the DSR for 2 menstrual cycles prior to intervention to confirm PMS diagnosis.	Intervention 1 (vitamin D group): 1 pill/d containing 200 mg of vitamin D Intervention 2 (vitamin E group): 1 pill/d containing 100 mg of vitamin E Placebo: 1 placebo pill/d (composition not stated) Length of intervention: 2 mo	Not stated. n = 36 of 86 participants completed the study	DSR	↔ Severity of total PMS symptoms
De Souza et al (2000)^40^	United Kingdom; randomized placebo-controlled, crossover	n = 44 women with PMS randomized to all 4 consecutive interventions for 1 cycle. Mean age: 32 y	Modified Menstrual Health Questionnaire assessed symptoms from the premenstrual phase of the participant's most recent menstrual cycle. Score >30% higher than postmenstrual total symptoms was assessed as PMS.	Intervention 1: 1 200 mg magnesium pill/d. Intervention 2: 1 50 mg vitamin B_6_ pill/d. Intervention 3: 1 combined 200 mg magnesium + 50 mg vitamin B_6_ pill/d. Placebo: 1 placebo pill/d (composition not stated). Length of intervention: 4 mo. No washout	Not stated. n = 37 of 44 participants completed the study	Daily menstrual diary using a 5-point ordinal scale with categories 0–4, corresponding to none, mild, moderate, severe, and very severe symptoms. n = 30 symptoms grouped into 6 categories: anxiety, craving, depression, hydration, other, and total	↓ Severity of anxiety-related premenstrual symptoms (nervous tension, mood swings, irritability, or anxiety) after combined magnesium + vitamin B_6_ intervention
Doll et al (1989)^41^	United Kingdom; cross-over RCT, double blinded	n = 63 women with PMS, aged 18–49 y	Participants with self-described PMS, reporting moderate to severe premenstrual symptoms during the previous year that were alleviated with the onset of menses	Intervention: 1 × 50 mg vitamin B_6_ pills/d. Placebo: 1 placebo pill/d (composition not stated) Length of intervention: 3 menstrual cycles for each treatment. No washout.	Not stated. n = 32 of 63 participants completed the study	Daily menstrual diary, which graded the severity of 9 individual symptoms from 0 to 3. Symptoms were divided into 3 categories: emotional (depression, irritability, tiredness); somatic; and menstrual.	↓ Severity of emotional symptoms (depression, irritability, and tiredness)
Esmaeilpour et al (2019)^42^	Iran; parallel RCT	n = 100 female nurses with PMS employed by private and state hospitals were randomized into intervention (n = 50) or control (n = 50) groups. Median age (IQR): intervention group: 34.5 y (IQR: 19, 45); control group: 27.5 y (IQR: 19, 45)	PMS diagnosis based on criteria of ACOG after 2 consecutive menstrual cycles were recorded via DSR, with participants reporting ≥1 of the following symptoms: depression, anger outburst, irritability, anxiety, confusion, social withdrawal, breast tenderness, abdominal bloating, headache, swelling of extremities.	Intervention: replacing at least 4 servings of refined grains with whole grains each day. 120 g of bread made with whole-grain flour (n = 4 servings) was supplied to the intervention group daily. Comparator: participants’ regular daily consumption of grains Length of intervention: 3 mo	Monthly follow-up via frequent telephone calls and face-to-face meetings 3-d food records and MET questionnaires (once before intervention then at each follow-up) Bread coupons were collected and counted. n = 76 of 100 participants completed the study	DSR-20 consisting of 20 different symptoms divided into 3 subgroups: mood, physical, and behavioral symptoms	↓ Severity of combined PMS symptoms
Facchinetti et al (1997)^43^	Italy; RCT, double blinded	n = 40 women with PMS referred by general practitioners or obstetricians were randomized into intervention (n = 20) or control (n = 20) groups. Mean age ± SD: intervention group: 28.0 ± 14.6 y; control group: 28.7 ± 14.9 y	PMS diagnosis confirmed by the prospective administration of MDQ, for 2 consecutive cycles, completed twice: 1 premenstrual period (days –8, –3), 1 follicular period (days +5, +11). MDQ consists of 47 symptoms experienced during each of 3 phases of the most recent menstrual cycle: (1) menses, (2) the week before menses, and (3) remainder of the cycle. Each symptom is scored from 1 to 6 on the Likert scale.	Intervention: 2 pills/d consisting of magnesium 400 mg, pyridoxine 1.5 mg, vitamin E 12 mg, folic acid 0.2 mg, iron 20 mg, copper 4 mg, *Saccharomyces cerevisiae* 1 g Placebo: 2 placebo pills/d (composition not stated) Length of intervention: 6 consecutive menstrual cycles (∼6 mo)	Not stated. n = 35 of 40 participants completed the study	MDQ completed twice during each menstrual cycle of the 2nd, 4th, and 6th mo of treatment: once in the premenstrual period (days –8, –3) and once in the follicular period (days +5, +11)	↓ Severity of combined PMS symptoms at each time observation (every 2nd month) ↓ Severity of combined PMS symptoms to 18% of baseline compared with placebo, by end of treatment (6th month)
Ghanbari et al (2009)^44^	Iran; RCT, double blinded	n = 179 female university students with PMS were randomized into intervention (n = 91) and control (n = 88) groups. Mean age ± SD: 21.4 ± 3.6 y (intervention and control groups)	A primary questionnaire was used to assess PMS diagnosis based on the Beck test, and the intensity of the symptoms was scored from 0 to 3. PMS symptoms included breast tenderness, fatigue, lack of energy, appetite changes, sleep problems, headache, depression, agitation, and irritability.	Intervention: 2 × 500 mg of calcium carbonate pills/d Placebo: 2 × 500 mg placebo pills/d (composition not stated) Length of intervention: 3 mo	Participants contacted once every week to encourage compliance. Contact method not stated. Completion rate not stated.	A secondary questionnaire was used to assess changes in severity of PMS symptoms based on the Beck test; the intensity of the symptoms was scored from 0 to 3. The PMS symptoms evaluated were as for the primary questionnaire.	↓ Severity of early tiredness, appetite changes, and depressive symptoms
Hagen et al (1985)^45^	Norway; crossover RCT, double blinded	n = 42 women with PMT. Mean age: 37 y (range, 24–46 y).	Authors interviewed participants and assessed PMS as regularly occurring problems commonly accepted as belonging to PMS, including irritability, breast tenderness, bloating, depression, headache, fatigue, acne, pain, hot flushes	Intervention: 1 × 100 mg vitamin B_6_ pill/d Placebo: 1 × 100 mg placebo pill/d (composition not stated) Length of intervention: 2 menstrual cycles for each treatment. No washout.	Compliance was evaluated by counting the number of tablets remaining at the end of the intervention period. n = 34 of 42 participants completed the study	PMS symptoms were scored on a VAS ranging from “no complaints,” to “maximal complaints.” Participants also ranked the severity of 6 symptoms (fatigue, headache, depression, irritability, breast tenderness, and bloating/weight gain).	↔ Severity of PMS symptoms
Heidari et al (2019)^46^	Iran; Parallel RCT, double blinded	n = 44 female university students with PMS were randomized into intervention (n = 22) and control (n = 22) groups. Mean age ± SD: 21.3 ± 1.6 y (intervention group), 21.7 ± 1.8 y (control group). All participants were vitamin D deficient (25(OH)D < 20 ng/mL) and had BMI between 18.5 and 25 kg/m^2^.	DSR for 2 consecutive menstrual cycles using a 4-point Likert scale (0 = none, 4 = severe). PMS cases were diagnosed based on the *DSM-IV* criteria.	Intervention: 1 pill of 50 000 IU vitamin D_3_ fortnightly Placebo: 1 pill containing edible paraffin fortnightly Length of intervention: 4 mo	Not stated. n = 38 of 44 participants completed the study	PMS symptoms were measured via DSR in the last 2 mo of the intervention.	↓ Severity of total PMS symptoms
Jafari et al (2020)^47^	Iran; RCT, double blinded	n = 60 female university students with PMS and BMI 18.5–24.9 kg/m^2^ were randomized into intervention (n = 30) and control (n = 30) groups. Mean age ± SD: intervention group: 23.04 ± 2.97 y; control group, 22.53 ± 1.85 y	Participants completed a 30-item checklist of PMS symptoms based on *DSM-IV*, for 2 consecutive menstrual cycles prior to the intervention. Those that experienced 5 of 30 PMS symptoms that interfered with their daily life, 7 d before menses until a maximum of 4 d after commencement of menses, were diagnosed with PMS.	Intervention: 1 zinc gluconate (containing 30-mg elemental zinc) pill/d Placebo: 1 × placebo pill/d (composition not stated) Length of intervention: 12 wk	Compliance with taking supplements and placebos was checked on return of empty tablet package and sending a short message service every day. n = 57 of 60 participants completed the study	Daily 30-item checklist of PMS symptoms based on *DSM-IV*	↓ Severity of combined physical and psychological PMS symptoms
Jones et al (1987)^48^	United States; RCT, matched-pair design	n = 37 women with PMS were paired based on relative weight (weight/height), then randomized to either a high-fat (40% energy from fat) or low-fat (20% energy from fat) diet containing 1 of 2 fatty acid ratios (polyunsaturated to saturated fatty acid ratios = 1:1 or 0.3:1).	Not stated	Intervention: high polyunsaturated to saturated fatty acid ratio (1:1); comparator: low polyunsaturated to saturated fatty acid ratio (0.3:1) All meals were provided and no other food outside the study was permitted. Length of consumption: n = 4 menstrual cycles for each treatment. No washout.	Not stated. n = 30 of 37 participants completed the study	MDQ administered at baseline, at the end of the high-fat diet period, and at the end of the low-fat diet period	↔ Severity of PMS symptoms
Kendall et al (1987)^49^	United States; RCT, double blinded, matched-pair design	n = 74 women with PMS were paired based on age and symptomology and randomized to receive vitamin B_6_ and control. Mean age ± SD: intervention group: 28.1 ± 6.5; control group: 27.7 ± 6.6	Premenstrual assessment form required participants to rate the premenstrual phase of the past 3 cycles and the degree of symptom change from normal levels	Intervention: 3 × 50 mg pill/d vitamin B_6_ Placebo: 3 × 50 mg pill/d placebo Length of intervention: 2 mo	Interviewed by study assistants once per cycle. Unused pills were returned and counted. n = 55 of 74 participants completed the study	Participants rated PMS symptoms every other day for 3 cycles using the MDQ	↓ Severity of negative behavioral symptoms (eg, poor performance, decreased social activities)
London et al (1991)^50^	United States; RCT, double-blinded.	n = 60 women with PMS were randomly allocated to 1 of 3 groups: placebo (n = 20), Optivite-6 (n = 20), or Optivite-12 (n = 20)	Modified version of MSQ was used to assess PMS. Symptoms were classified into 4 categories: PMT-A (anxiety), PMT-H (fluid retention), PMT-C (cravings), and PMT-D (depression). Severity of symptoms was rated on a 4-point Likert scale. PMS was defined as ≥1 category being absent or mild during the midfollicular phase, but moderate to severe during the late luteal phase.	Intervention 1 (Optivite-6^a^): 3 × multivitamin and 3 × placebo tablets twice daily (n = 6 multivitamin and 6 placebo tablets/d) Intervention 2 (Optivite-12^a^): 6 × multivitamin tablets twice daily (total, 12/d) Placebo: 6 × placebo tablets twice daily with meals (total, 12/d). Length of intervention: 3 mo	Compliance was assessed by counting returned tablets, as well as with phone calls to participants. n = 56 of 60 participants completed the study. n = 44 of 60 were included in the final analysis	The MSQ was completed by participants during the midfollicular and late luteal phases of each cycle.	Failed to report between-group differences
Penland et al (1993)^51^	United States; double-blinded, crossover	n = 14 women residing 24 h/d in a metabolic research ward at Human Nutrition Research Center were randomly assigned to each of 4 × 39-d dietary periods. Mean age ± SD: 27.2 ± 1.6 y	Recruited participants reported normal menstrual patterns with no history of dysmenorrhea or current symptoms of premenstrual tension.	Participants were provided a conventional American adult diet (energy intakes between 1600 and 2800 kcal/d), with calcium and manganese altered by researchers. Women were provided 800 mg calcium/d and 2.97 mg manganese/d for 13-d baseline period. No washout. Intervention 1: 587 mg calcium + 1.0 mg manganese/d Intervention 2: 1336 mg calcium + 1.0 mg manganese/d Intervention 3: 587 mg calcium + 5.6 mg manganese/d Intervention IIII: 1336 mg calcium + 5.6 mg manganese/d Length of intervention: 169 d (with each participant completing 4 × 39-d dietary periods, as well a 13-d baseline period)	Participants resided in a metabolic research ward for strict control of diet, exercise, and data collection, as well as to provide a common environment. Participants were chaperoned on all outings to ensure compliance with dietary protocol. n = 14 of 14 participants completed the study	MDQ was administered to participants at the end of each menstrual cycle. Symptoms were grouped into 8 categories: autonomic reactions, behavior change, concentration, negative affect, pain, water retention, arousal, and control.	↓ Negative affect and behavior change for high-calcium diets compared with low-calcium diets for all menstrual phases ↓ Concentration of high-calcium compared with low-calcium diet for premenstrual phase. ↔ Arousal, autonomic reactions, and control between dietary groups
Retallick-Brown et al (2020)^52^	New Zealand; treatment-controlled trial, double-blind	n = 78 women with PMS were randomly allocated to receive vitamin B_6_ (n = 37) or a micronutrient (n = 41) supplement. Mean age ± SD: B_6_ group: 34.32 ± 10.17 y, micronutrients group: 36.16 ± 7.92 y	Women were followed for 2 menstrual cycles at baseline and completed a modified version of the DRSP questionnaire. For a diagnosis of PMS, this 14-item questionnaire requires ≥1 symptom and a minimum of 3 symptoms (out of 11) to worsen during the luteal phase of the cycle and only mild symptoms during the midfollicular phase, and have a negative impact on quality of life. Symptoms were rated on a Likert scale from 1 to 6. The DRSP produced 5 measured outcomes (psychological, somatic, total, impact ratings, and worst day ratings).	Intervention: 4 × EMP+ capsules^b^ twice/d Comparator: 4 × vitamin B_6_ capsules (containing 80 mg B_6_, 0.8 mg riboflavin) twice/d. Length of intervention: 3 mo.	Participants were reminded at monthly meetings to complete the questionnaire, and daily reminders (via email or text) were sent during the luteal phase. Capsule consumption was counted and <80% consumption was considered noncompliant with treatment protocol. n = 72 of 78 participants completed intervention	Participants completed the DRSP daily for 2 baseline cycles and 3 treatment cycles as the primary outcome. The DASS-42 was used to measure negative emotional states, and the PSS-10 was used to measure daily stress. The WQoLQ was used to assess quality of life in physical, psychological, social, and spiritual domains.	↔ DRSP scores at end of treatment between B_6_ and EMP+ group ↔ DASS-42 and PSS-10 between B_6_ and EMP+ group after treatment ↑ WQoLQ for EMP+ group compared to B_6_ after treatment
Rocha Filho et al (2011)^53^	Brazil; RCT, double-blinded	n = 120 women with PMS were randomly allocated to 3 groups: low-dose FAs (n = 40), high-dose FA (n = 40), or placebo (n = 40). Mean age ± SD: low-dose FA group: 33.0 ± 6.6 y; high-dose FA group: 32.4 ± 6.1 y; placebo group: 32.7 ± 6.3 y	Women completed the PRISM calendar for 2 consecutive months to determine the presence of PMS. The PRISM calendar measures 23 physical symptoms and participants are required to score each symptom between 0 (no experienced) and 3 (severe) daily. At the end of the month, scores for the follicular and the luteal phase are totaled separately. Participants were considered to have PMS if there was a minimum 30% increase in scores between the follicular and luteal phases.	Intervention 1 (low-dose FA): 1 × 1 g FA capsule (containing 210 mg γ-linolenic acid, 175 mg oleic acid, 345 mg linoleic acid, 250 mg other PUFAs, 20 mg vitamin E) + 1 placebo capsule/d Intervention 2 (high-dose FA): 2 × 1 g FA capsules/d Placebo: 2 × placebo capsules/d (containing 1 g mineral oil) Length of intervention: 6 mo	Blister packs of supplements (whether all supplements were taken or not) were returned to investigators at each clinic visit monthly, and participants were questioned about their compliance. n = 116 of 120 participants completed the study	The PRISM calendar was completed daily by participants and total scores were calculated for the follicular and luteal phases of the menstrual cycle.	↓ Median total PRISM scores for both 1 g and 2 g treatment groups at 3 mo and 6 mo ↓ Median total PRISM score for 2 g group compared with 1 g group at 3 and 6 mo
Sadeghi et al (2022)^54^	Iran; RCT, double-blinded	n = 50 female students staying in medical university dormitories were randomly allocated to the intervention (n = 25) or control (n = 25) groups. Mean age ± SD: intervention group: 28.4 ± 4.1 y; control group: 29.0 ± 3.9 y	The presence and severity of PMS was assessed using a symptom questionnaire. Mild PMS is classified as a score of 2–16, moderate as a score of 17–25, severe as a score of 26-33, and very severe as a score >33. Women with moderate to severe PMS were recruited for the next screening phase. These women then recorded their daily menstrual symptoms for 2 cycles using a 22-item questionnaire on a 0–3 Likert scale. Participants scoring moderate to severe were included in the study.	Intervention: 2 × 550 mg PMS50^c^ supplements/d. Placebo: 2 × placebo tablets/d. Length of intervention: 3 menstrual cycles (∼3 mo), but only took the tablets from 1 wk before menstruation to the end of menstruation	Not stated n = 46 of 50 participants completed the study	The MSQ was used to record daily symptoms. Dietary intakes of macro- and some micronutrients (B_1_, B_2_, B_6_, E, D_3_, and Mg) were assessed using a 3-d food recall questionnaire at the beginning and end of intervention.	↓ Total PMS scores after 1, 2, and 3 mo of treatment ↓ Restlessness, depression, anger, irritability, anxiety, mood swings, hopelessness, desire to be alone, and poor concentration, after 3 mo of treatment
Samieipour et al (2016)^55^	Iran; RCT, single-blinded	n = 264 female students with PMS residing in the Ilam University dormitories were randomly allocated to 4 treatments: vitamin B_1_ (n = 66), calcium carbonate (n = 66), vitamin B_1_ + calcium carbonate (n = 66) and placebo (n = 66). Mean age ± SD: 20.3 ± 8.9 y	PMS was confirmed in participants through a PMS diagnosis questionnaire designed using the *DSM-IV* criteria of PMDD. Participants completed a 21-item questionnaire on the severity and frequency of symptoms (11 psychiatric and 10 physical). Participants were asked to record their symptoms for 2 wk prior to menstruation for the previous 2 mo. A minimum of 5 symptoms, 7 d prior and up to 4 d after menstruation indicated the presence of PMS.	Intervention 1: 1 × 100 mg vitamin B_1_ tablet/d Intervention 2: 1 × 500 mg calcium carbonate tablet/d Intervention 3: 1 × 100 mg vitamin B_1_ and 1 × 500 mg calcium carbonate/d. Placebo: 1 × 1 g starch tablet/d. Length of intervention: 2 menstrual cycles (∼2 mo), but only took tablets 1 wk before menstruation to 4 d after menstruation	Not stated n = 239 of 264 participants completed the study	Mean PMS symptoms and severity were measured using the PMS diagnosis questionnaire used to screen participants. Questionnaires were completed for 2 menstrual cycles during the 1 wk before menstruation and 4 d after menstruation.	↓ Mean PMS symptoms for all intervention groups
Sayegh et al (1995)^56^	United States; double-blinded, crossover	n = 99 women with PMS were randomly allocated to 1 of 3 treatment groups: beverage A, beverage B, and beverage C. Mean age ± SD: 37 ± 1.2 y	Participants were required to meet the criteria of the National Institute of Mental Health for PMS, meaning a 1-y history of ≥5 symptoms, with ≥1 of depression, affective lability, tension, or irritability. Symptoms were required to be present during premenstrual phase, impair daily activities, and subside with onset of menses. Participants rated symptoms (mood, appetite, work/social impairment, physical symptoms) for 2 cycles using a symptom rating scale.	Intervention 1 (beverage A): 47.5 g mixture of dextrose and maltodextrin, reconstituted in water, consumed during 1 d of late luteal phase of the cycle. Intervention 2 (beverage B): 15 g casein and 32.5 g dextrose in water, consumed during 1 d of late luteal phase of cycle. Intervention 3 (beverage C): 47.5 g mixture of galactose and dextrose in water, consumed during 1 d of late luteal phase of cycle. Length of intervention: 3 mo	Guidelines regarding the size and type of meal participants were allowed to eat were provided to minimize the effect of food on results. Participants were not allowed to consume any food or drink, apart from water, during the testing period. n = 24 of 99 participants completed the study	Mood and appetite scores were obtained immediately before consuming drink, and at 30, 90, and 180 min after consuming drink. Mood was measured using POMS, which rated tension, depression, anger, and confusion on a 5-point Likert scale. A 10-point Likert scale was used to rate cravings for protein-rich, fat-rich, and carbohydrate-rich foods, as well as fruits, vegetables, and general appetite. Participants also completed 3 cognitive function tests at 90 min.	↓ Total POMS score, anger, carbohydrate craving for beverage A at 180 min after treatment compared with baseline and with drinks B and C ↓ Depression with beverage A compared with beverage B at 180 min after consumption
Siahbazi et al (2017)^57^	Iran; RCT, double-blinded	Women who had sought treatment for PMS at a gynecology clinic were eligible to participate. n = 142 women with PMS were randomly allocated to either receive a zinc supplement (n = 71) or placebo (n = 71). Mean age ± SD: zinc group: 22.4 ± 3 y; placebo group: 22.6 ± 2.2 y	PMS was defined as the recurrence of behavioral, psychological, or somatic symptoms during the premenstrual phase of the menstrual cycle for at least 4 cycles out of the last 6. The PSST (Iranian version) was used to identify the presence and severity of PMS. The SF-12 survey (Iranian version) was also used to assess general physical and mental health.	Intervention: 1 × 220 mg zinc sulfate capsule (containing 50 mg elemental zinc)/d. Placebo: 1 × 220 mg sucrose capsule/d Length of intervention: 3 menstrual cycles (∼3 mo), starting on the 16th day of the cycle (midluteal phase) to the second day of the next cycle.	Participants recorded medication compliance and any side effects. Assessed by monthly follow-up visits at the end of menstruation when questionnaires were collected, and new medications were dispensed. n = 130 of 142 participants completed the study	Participants were asked to complete the PSST and SF-12 survey on days 7–10 of the menstrual cycle. Primary outcome was mean rank symptoms and severity measured on the PSST for the intervention group vs control group.	↓ All symptoms and components measured on PSST in zinc group at end of 3 mo ↑ Physical and mental components of SF-12 survey for zinc group after 3 mo
Stewart (1987)^58^	United Kingdom; RCT, double-blinded	High-dose trial: n = 175 women with PMS were randomly allocated to receive a placebo (n = 100) or supplement (n = 75) capsule. Low-dose trial: 143 women were allocated to placebo (n = 75) or supplement (n = 68).	n = 19 PMS symptoms were assessed by a questionnaire and categorized into 4 groups: PMT-A (anxiety), PMT-H (fluid retention), PMT-C (cravings), PMT-D (depression). Each symptom was rated on a 4-point Likert scale for 1 wk after menses (follicular phase) and 1 wk prior (luteal phase). The luteal score minus the follicular score gave an approximation of the PMS severity. Women were included if they had scores ≥4 for PMT-A, ≥4 for PMT-H, ≥6 for PMT-C, and ≥6 for PMT-D, as well as ≥1 PMT subgroup.	Intervention 1 (high-dose): 4 × Optivite/d^a^ (2 in the morning, 2 in the evening) for first 2 wk of each cycle, increasing to 8 × Optivite/d^a^ (n = 4 in the morning, n = 4 in the evening) for the last 2 wk of each cycle. Intervention 2 (low dose): 2 × Optivite/d^a^ (1 in the morning, 1 in the evening) for first 2 wk, then 4 × Optivite/d^a^ for last 2 wk of cycle. Placebo: 1 × placebo capsule/d (containing 500 mg mineral oil) Length of intervention: high-dose trial: 3 mo; low-dose trial: 4 mo	Women were monitored by telephone and/or letter at 2- to 4-wk intervals. n = 119 of 175 women completed the high-dose trial. n = 104 of 143 women completed the low-dose trial	Women completed a questionnaire assessing PMS symptoms at the beginning and end of treatment period. Women were also asked at the end of the study to decide whether symptoms had become worse, no better, slightly better, substantially better, or cured.	Failed to report between-group differences
Tartagni et al (2016)^59^	Italy; RCT, single-blinded	n = 164 adolescents (aged 15–21 y) with PMS and severe vitamin D deficiency (<10 ng/mL) were randomly divided into 2 groups: intervention group (n = 82) and control group (n = 82). Mean age ± SD: intervention group: 19.8 ± 1.4 y; control group: 18.6 ± 1.9 y	Diagnostic criteria for PMS from ACOG (minimum 1 affective and 1 somatic symptom 5 d prior to menses for 3 previous menstrual cycles). Participants were screened for emotion and cognitive PMS symptoms using DSR. Participants reporting severe and extremely severe symptoms qualified for the study.	Intervention: vitamin D_3_ (initial dose of 100 000 IU, followed by 25 000 IU every 2 wk for 4 mo) Placebo: composition not stated Length of intervention: 4 mo	Not stated. n = 158 of 160 participants completed the study	Completion of the DSR for length of intervention	Failed to report between-group differences
Thys-Jacobs et al (1989)^60^ and Alvir and Thys-Jacobs (1991)^61^	United States; cross-over RCT, double blinded	n = 60 women, of whom most were employees at a New York hospital, were enrolled into the study. Mean age ± SD: 35.1 ± 6.0 y	To assess PMS, participants completed a DSR. Women who qualified for the study had a minimum 50% increase in intensity of symptoms during the luteal phase compared with the intermenstrual phase.	Intervention: 2 × calcium carbonate tablets/d (1000 mg elemental calcium/d) Placebo: 2 × placebo tablets/d (composition not stated) Length of intervention: 3 menstrual cycles for each treatment. No washout.	Compliance assessed every 4 wk by counting remaining tablets and through biweekly contact by research nurses. 70% compliance was required to be included in the study. 1989 study^60^: n = 33 of 60 participants completed the study. 1991 study^61^: n = 33 of 33 participants completed the study.	DSR scores for 14 PMS symptoms were totaled by investigators. A retrospective assessment was also made by participants about whether their symptoms improved during the first or second phase of the study. Principal component analysis identified 4 factors that explained 67% of the total variance in the data (negative affect, water retention, food/appetite, and pain).	↓ Total mean symptom scores during both luteal and menstrual phases ↓ Negative affect (depression, violent tendencies, crying, mood swings, irritability, nervousness) during luteal phase ↔ Negative affect, water retention, food cravings during menstrual phase
Thys-Jacobs et al (1998)^62^	United States; parallel RCT, double-blinded	n = 497 women were randomized into 2 treatment groups: calcium supplementation (n = 248) and placebo (n = 249). Mean age ± SD: 32.8 ± 6.7 y	Participants were screened for PMS by a clinician who evaluated the impact of PMS on participants' lives. Participants also completed the PMS Diary, a daily self-assessment questionnaire for 2 menstrual cycles. The PMS Diary comprises 17 items measuring negative affect, water retention, food cravings, and pain using a 4-point Likert scale.	Intervention: 4 × 750 mg calcium carbonate pills (containing 300 mg elemental calcium each)/d Placebo: 4 × placebo pills/d Length of intervention: 3 menstrual cycles (∼3-mo)	Participants were contacted via telephone biweekly and monthly follow-up visits on days 7–10 of the menstrual cycle. Compliance was assessed at each follow-up visit; unused treatment pills were collected and counted at the end of the study. 80% compliance was required for analysis. n = 466 of 497 participants completed the study	Primary outcome was the symptom complex score, which was calculated from the 17 symptoms measured in the PMS Diary. Efficacy was measured as the difference between the symptom complex score on calcium treatment compared with placebo.	↓ Mean symptom complex score during luteal phase of cycle 1 + 2. ↓ Negative affect and food cravings by cycle 3 ↓ Individual symptom scores (mood swings, depression, tension, anxiety, anger, crying spells, cravings, appetite changes) by cycle 3
Walker et al (1998)^63^	United Kingdom; crossover RCT	n = 54 women with PMS were recruited from the University of Reeding. Participants who completed the study were aged between 18 and 50 y.	Before commencing the study, participants were asked to complete the MHQ, which was divided into 2 sections. One section investigated participants' general health and previous PMS symptoms. The other section was a 27-item, 5-point Likert scale retrospective assessment of the severity of PMS symptoms experienced during the previous cycle.	Intervention: 1 mg tablet (containing 200 mg magnesium oxide and 100 mg mixed amino acids)/d Placebo: 1 placebo tablet (containing microcrystalline cellulose)/d. Length of intervention: 4 mo (2 mo of each treatment). No washout.	Urine was collected and compliance to treatment was assessed as a significant increase in urinary Mg excretion. n = 38 of 54 participants completed the first 2 treatment cycles	Participants kept a daily menstrual diary of their symptoms based on the MDQ, using a 4-point Likert scale. 22 symptoms were grouped for analysis: PMS-A (anxiety, irritability), PMS-C (craving, headache, increased appetite), PMS-D (depression, insomnia, forgetfulness), PMS-H (hydration, weight gain, swelling), and PMS-O (other, cramps, pain).	↔ PMS-A, PMS-C, PMS-D, PMS-O for both months ↔ PMS-H during first month ↓ PMS-H during second month
Wurtman et al (1989)^64^	United States; crossover RCT	n = 18 women with PMS and 14 control participants who were age- and weight-matched	Potential participants completed a health history questionnaire evaluating changes in mood, appetite, sleep, and somatic symptoms during the follicular and luteal stages of their menstrual cycle. During the late luteal phase of their menstrual cycle, potential participants underwent a structured psychiatric interview, including the Hamilton Depression Scale and a modified addendum assessing fatigue, carbohydrate cravings, appetite, and sociability.	High-carbohydrate, low-protein meal containing 561 calories, 112 g carbohydrate, 6 g protein, and 16 g fat. Meal was given to participants once during the early follicular phase and once during the late luteal phase. Length of intervention: 2 × single identical meals (1 meal consumed during early follicular phase and 1 meal consumed during late luteal phase)	Meal was provided at the research center. All food provided was weighed in containers before it was served, and the containers were reweighed after the meal was completed. n = 32 of 32 participants completed the study	The POMS is a questionnaire that measures 6 factors: tension-anxiety, depression-dejection, vigor-activity, anger-hostility, fatigue-inertia, and confusion-bewilderment. The Visual Analogue Mood Scale is a self-reported questionnaire used to measure 3 mood states: alert, sad, calm.	Failed to report between-group differences
Yilmaz-Akyuz et al (2019)^65^	Turkey; RCT, single-blind	First-year female students in the Faculty of Health Sciences at a public university were randomly assigned to exercise (n = 37), diet (n = 37), and control (n = 37) groups. Mean age ± SD: 19.4 ± 1.6 y; mean BMI: 21.3 ± 2.8 kg/m^2^	All potential participants were followed up for 2 menstrual cycles to determine the presence of PMS, using the PMSS, a 5-point Likert scale with 44 items. The PMSS has 9 subscales: depressive mood, anxiety, tiredness, anger bursts, depressive thoughts, pain, appetite changes, sleep disturbances, and abdominal bloating. A higher score than the maximum score on the subscale is indicative of PMS.	Intervention 1 (PMS diet): 50%–55% carbohydrates, 15%–20% protein, 25%–30% fat. Diet rich in complex carbohydrates, fish consumed 1–2 timers per week. Daily consumption of >1000 mg calcium, 20 g dried nuts, <300 mg caffeine. Refined sugar and added salt were limited. Intervention 2 (exercise): aerobic exercise was performed for 30 min, 3 d/wk Comparator: usual diet or physical activity Length of intervention: 12 wk	Diet: research dietitian had telephone or face-to-face interviews with individuals once a week. Exercise: conducted at a sports center with an experienced trainer. n = 106 of 111 participants completed the study	PMS symptoms were measured using the PMSS. An FFQ containing 10 items was used to assess dietary intake.	↔ Mean total PMSS scores
Yurt et al (2020)^66^	Turkey; RCT	n = 40 women studying at the Eastern Mediterranean University, aged between 20 and 28 y, were diagnosed with PMS and were consuming less than the RDA of calcium were randomized into intervention (n = 20) and control (n = 20) groups.	Participants were previously diagnosed with PMS by a doctor.	Intervention: consumption of RDA of 1000 mg calcium (70%–80% from dairy, 20%–30% from other food groups) for 2 mo, including 50 g Turkish *kasseri* cheese, 400 mL milk, and 150 g yoghurt/d Comparator: Consumed usual diet. Length of intervention: 2 mo	Participants in the intervention group were provided 50 g Turkish *kasseri* cheese daily. Energy, macro-, and micronutrients intake from the 3-d food record was used to ensure participants were meeting the RDA for calcium. Data from 31 of 40 participants were analyzed.	PMS was measured using the PMSS. The SF-36 tool was also administered. Both scales were completed at the beginning and end of the study.	↓ Depressive mood, anxiety, tiredness, depressive thoughts, sleep disturbances, anger, and total PMSS scores in dairy group ↑ Mental health on SF-36 in dairy group

a Composition of 12 Optivite tablets: 25 000 IU vitamin A, 200 IU vitamin E, 200 IU vitamin D_3_, 400 μg folic acid, 50 mg vitamin B_1_, 50 mg vitamin B_2_, 50 mg niacinamide, 600 mg vitamin B_6_, 125 μg vitamin B_12_, 50 mg pantothenic acid, 625 mg choline bitartrate, 3000 mg vitamin C, 500 mg magnesium, 150 μg iodine, 30 mg iron, 1.0 mg copper, 50 mg zinc, 20 mg manganese, 95 mg potassium, 200 μg selenium, 200 μg chromium.

b Composition of EMP+ per 8 capsules: 3072 IU vitamin A, 320 mg vitamin C, 768 IU vitamin D, 192 IU vitamin E, 9.6 mg thiamin, 7.2 mg riboflavin, 48 mg niacin, 19.2 mg vitamin B_6_, 768 μg folic acid, 480 μg vitamin B_12_, 576 μg biotin, 11.52 mg pantothenic acid, 704 mg calcium, 7.34 mg iron, 448 mg phosphorous, 108.8 μg iodine, 320 mg magnesium, 25.6 mg zinc, 108.8 μg selenium, 3.84 mg copper, 5.12 mg manganese, 332.8 μg chromium, 76.8 μg molybdenum, 128 mg potassium.

c Composition of daily dose of PMS50 supplement: 1.4 mg vitamin B_1_, 1.6 mg vitamin B_2_, 2 mg vitamin B_6_, 10 mg vitamin E, 5 μg vitamin D_3_, 300 mg magnesium oxide, 0.1 g dried *Vitex agnus* extract.

*Abbreviations:* ACOG, American College of Obstetricians and Gynecologists; BMI, body mass index; DASS-42, 42-item Depression Anxiety and Stress Scale; DRSP, Daily Record of Severity of Problems; *DSM-IV*, *Diagnostic and Statistical Manual of Mental Disorders* (Fourth Edition); DSR, daily symptom record; EMP+, EMPowerPlus Advanced; FA, fatty acid; FFQ, food frequency questionnaire; IQR, interquartile range; MDQ, Menstrual Distress Questionnaire; MET, metabolic equivalent of task; MHQ, Menstrual Health Questionnaire; PMS, premenstrual syndrome; PSST, Premenstrual Symptoms Screening Tool; PMSS, Premenstrual Syndrome Scale; PMT, premenstrual tension; POMS, Profile of Mood States; PRISM, Prospective Record of the Severity of Menstruation; PSS-10, 10-item Perceived Stress Scale; PUFA, polyunsaturated fatty acid; RCT, randomized controlled trial; RDA, recommended dietary allowance; SF-12, 12-Item Short Form Health Survey; SF-36, 36-Item Short Form Health Survey; WQoLQ, Womens’ Quality of Life Questionnaire; VAS, Visual Analog Scale; ↓, significantly lower than that in the comparison control group after intervention; ↑, significantly higher than that in the comparison control group after intervention; ↔, no significant difference between the intervention and control groups after intervention.

### Risk of bias

**Figure 2:**
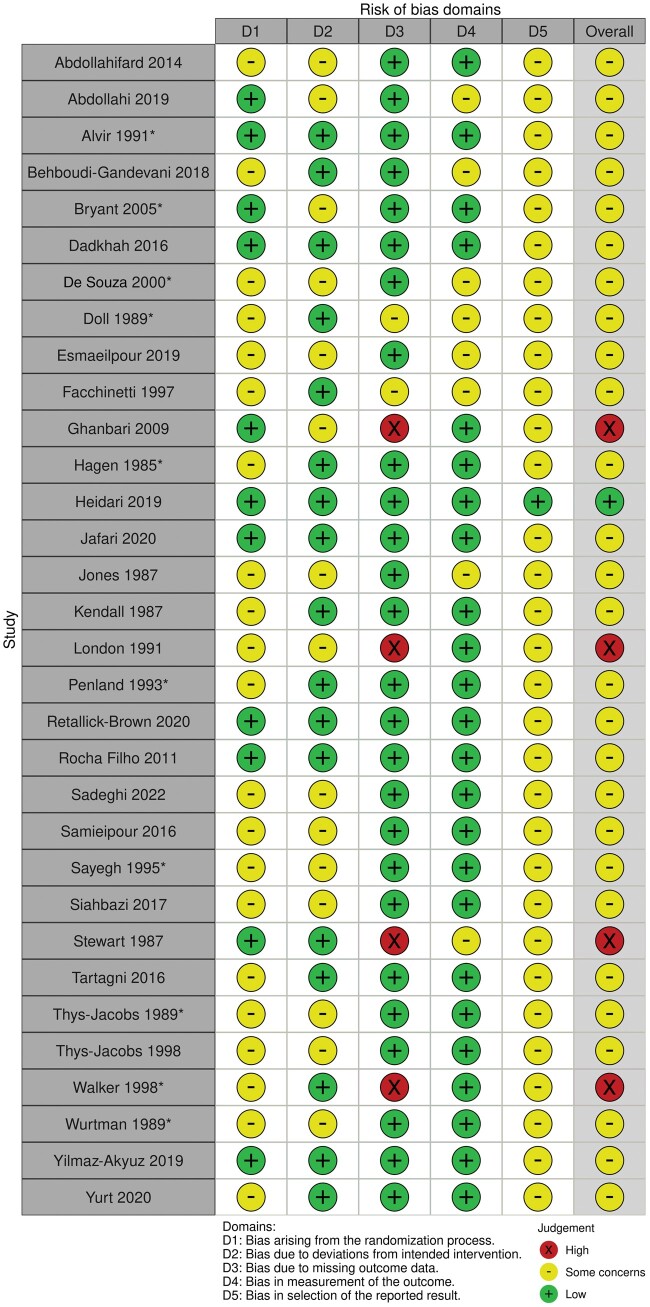
**Risk-of-bias assessment for randomized studies using the Cochrane Risk of Bias 2 (RoB 2) tool (traffic light plot)**. The “x” symbol in the traffic light plot indicates high risk of bias, the “–” symbol indicates some concerns, and the “+” symbol indicates low risk of bias. *Indicates crossover trials, which were assessed using the RoB 2 tool for crossover trials.

Figure 2 summarizes risk-of-bias assessments for included articles. Using the RoB2 tool for assessment of RCTs or for crossover trials, 4 of the studies (13%) included in the present review were classified as being at high risk of bias, 27 (84%) had some concerns, and only 1 study (3%) was designated as low risk of bias. Almost all studies (97%) had some concerns relating to bias of the selection of reported results (because most authors had not prepublished their statistical analysis plan in a clinical trial registry), and 66% had some concerns of bias arising from an unclear randomization process. Four studies (13%) were classified as having a high risk of bias due to high study attrition rates with no reasons provided why participants discontinued their involvement in the trials.

### Effects of nutritional interventions on premenstrual syndrome symptoms

#### Vitamin supplementation

Eight studies (n = 719 participants) investigated the impact of vitamin supplementation on psychological PMS symptoms, 4 of which examined B-group vitamins and 4 studies examined vitamin D (Table 2).^35^^,^^36^^,^^39^^,^^41^^,^^45^^,^^46^^,^^49^^,^^59^ A single parallel RCT with some concerns of bias involving 100 Iranian university students reported a 35% average decrease (*P* < 0.001) in combined psychological PMS symptoms after a 2-month intervention of 200 mg/d vitamin B_1_ (thiamin).^35^ Two RCTs (n = 137 participants) demonstrated that 50 mg/d vitamin B_6_ (pyridoxine) for 3 months reduced emotional symptoms (depression, irritability, and tiredness) when compared with those receiving a placebo (*P* < 0.05),^41^ and 150 mg/d vitamin B_6_ for 2 months significantly improved behavioral symptoms (namely, poor performance and withdrawal from social activities) (*P* < 0.05).^49^ In contrast, a crossover RCT involving 34 Norwegian women who received 100 mg/d vitamin B_6_ for 2 menstrual cycles reported the intervention did not have an effect on PMS symptoms.^45^ Limitations of these studies included retrospective self-reporting of symptoms for PMS diagnosis (rather than analysis of symptom records collected prospectively over at least 2 menstrual cycles)^41^^,^^45^ and low study retention rates.^41^^,^^49^

Three studies (n = 354 participants) investigated vitamin D supplementation (ranging from 2000 IU daily for 3 months to 50 000 IU fortnightly for 4 months) in participants with vitamin D deficiencies, using validated tools to measure PMS outcomes.^36^^,^^46^^,^^59^ One high-quality trial reported a significant reduction in total PMS symptoms in the treatment group (*P* < 0.001) in comparison with the control group,^46^ whereas another study with some concerns of bias found no significant differences between vitamin D supplementation and a placebo.^36^ The third study did not report differences between the intervention and control groups; therefore, study findings are inconclusive.^59^ Additionally, a smaller trial of 86 healthy participants (only 36 completed the study) using 200 mg/d vitamin D or 100 mg/d vitamin E for 2 months found no significant effects in the intervention group compared with the control group.^39^

Overall, vitamin B_6_ supplementation of at least 50 mg/d may have a positive effect on psychological PMS symptoms: a majority of trials identified significant improvements compared to a placebo. It remains unclear, however, whether vitamin D has any effect in women without preexisting vitamin D deficiency.

#### Minerals

A total of 8 RCTs (n = 1032 participants) examined the effects of dietary mineral consumption on psychological symptoms of PMS, including calcium, zinc, and magnesium (Table 2).^44^^,^^47^^,^^60–63^^,^^57^^,^^66^ All 4 trials (n = 776 participants) investigating calcium intake reported positive impacts on PMS symptoms. A crossover RCT reported that 2000 mg/d calcium for 3 months decreased the severity of total average PMS symptom scores and negative affect (namely, depression, violent tendencies, crying, mood swings, irritability, and nervousness) (*P* < 0.05).^60^^,^^61^ In a parallel study of 497 women, a calcium dose of 1200 mg/d for 3 months resulted in a 48% decrease in total PMS symptom scores (*P* < 0.001).^62^ Another trial found that 1000 mg/d calcium significantly reduced PMS-related early tiredness (*P* < 0.05), appetite changes (*P* < 0.01), and depressive symptoms (*P* < 0.01) after 3 months in university students.^44^ In contrast to supplementation, 31 Turkish university students with inadequate calcium intakes were randomized to consume either calcium-containing foods (70%–80% from dairy and 20%–30% from other food groups, equivalent to ≥1000 mg/d) or their usual diet for 2 months.^66^ Study findings indicated that sufficient calcium intake reduced total PMS symptoms (*P* < 0.001), individual psychological symptoms (ie, depressive mood, anxiety, tiredness, anger, and sleep disturbances) (*P* < 0.05), and improved quality of life (*P* < 0.05).^66^ Zinc supplementation of 30 mg/d for 3 months had beneficial effects on physical and psychological PMS symptoms in 60 university students (*P* < 0.01),^47^ and 50 mg/d zinc supplementation for 3 months improved mental health scores and quality of life in 142 Iranian women with PMS (*P* < 0.05).^57^ A crossover RCT involving 54 women from the United Kingdom did not find a significant impact of 200 mg/d magnesium supplementation over 2 months on psychological PMS symptoms.^63^ In summary, evidence suggests that at least 1000 mg/d calcium from either nutritional supplements or foods, and zinc supplementation of at least 30 mg/d may be effective in reducing emotional PMS symptoms and improving quality of life.

#### Multivitamin and mineral supplementation

Eight studies (n = 725 participants) in which combined nutrient or multivitamin supplementation for psychological PMS symptoms was investigated were included in this review (Table 2).^40^^,^^43^^,^^50–52^^,^^54^^,^^55^^,^^58^ Of the 5 RCTs investigating multivitamins,^43^^,^^50^^,^^52^^,^^54^^,^^58^ 2 used Optivite (Optimox Corporation, Torrance, California, USA) (a multivitamin and mineral supplement marketed specifically to women with PMS), produced inconclusive results due to use of nonvalidated outcome assessment tools^58^ and failure to report between-group differences.^50^^,^^58^ Both studies had a high risk of bias, and 1 was funded by the manufacturer of Optivite.^50^

The remaining 3 multivitamin studies produced mixed results. A yeast-containing multivitamin significantly reduced combined PMS symptoms compared with placebo (*P* < 0.05),^43^ decreasing symptoms to 18% and 75% of baseline, respectively. This supplement also contained a variety of other vitamins and minerals (including 500 mg/d magnesium), so it is uncertain what component of the supplement was contributing to the improved symptoms.^43^ A parallel study using PMS50 (Pharmalife Research, Lombardy, Italy), a supplement specifically designed for PMS and containing vitamins, minerals, and plant extracts, reported a reduction in total PMS symptoms (*P* < 0.001), including specific psychological symptoms such as depression, restlessness, anger, irritability, anxiety, mood swings, hopelessness, and poor concentration.^54^ Conversely, another PMS-specific multivitamin and mineral supplement, EMPowerPlus Advanced (Truehope Nutritional Support Ltd., Raymond, Canada), did not significantly reduce psychological symptoms when compared with 80 mg/d vitamin B_6_ supplementation, but it improved quality of life.^52^

Three studies investigated the effects of dual nutrient supplementation, including calcium, manganese, vitamin B_1_, and vitamin B_6_.^40^^,^^51^^,^^55^ Samieipour et al^55^ examined the effect of either 100 mg/d vitamin B_1_ alone, 500 mg/d calcium carbonate alone, or a combination of 100 mg vitamin B_1_ and 500 mg calcium carbonate on PMS symptoms in university students. Combined PMS symptoms were reduced in the vitamin B_1_ plus calcium intervention group after 2 treatment cycles (*P* < 0.001), but this study had some concerns regarding risk of bias. In a strictly controlled crossover study, 14 healthy participants were provided conventional American diets supplemented with high and/or low amounts of calcium and manganese over 4 intervention periods of 39 days each.^51^ The high-calcium diet significantly improved negative affect and behavior (*P* < 0.05) compared with the low-calcium diet. Despite being high in calcium, the low-manganese diet resulted in increased negative affect (*P* = 0.035), suggesting a possible synergy between the 2 minerals. In a randomized crossover study of 44 British women with PMS, the daily combination of 200 mg of magnesium and 50 mg of vitamin B_6_ for one month resulted in a significant decrease in anxiety-related symptoms (ie, nervous tension, mood swings, irritability, and anxiety) compared with single nutrients or placebo (*P* = 0.04).^40^

Overall, evidence for combined nutrient and/or multivitamin supplements to alleviate psychological PMS symptoms is limited. The optimal dose and composition of these supplements required for maximal symptom relief are unknown.

#### Dietary fatty acids

Three studies (n = 252 participants) analyzed the effect of dietary fatty acids on psychological symptoms of PMS.^37^^,^^48^^,^^53^ In a study involving 95 Iranian women aged 20–35 years, 2 g of omega-3 fish oil was given for 10 consecutive days per month (during the luteal phase) over 3 months and effectively reduced mental and behavioral symptoms (all *P* < 0.05).^37^ Rocha Filho et al^53^ randomized 120 Brazilian women into 3 parallel treatments: (1) 1 g/d polyunsaturated fatty acid supplement, (2) 2 g/d polyunsaturated fatty acid supplement, or (3) placebo, over 6 months. The 2 g/d dose of polyunsaturated fatty acid supplement significantly improved total PMS scores at 3 and 6 months compared with the 1 g/d dose (*P* < 0.003) and placebo (*P* < 0.001).^53^ In contrast, a smaller study of 30 women did not observe any significant improvement in psychological PMS symptoms after an 8-month trial comparing a high vs low polyunsaturated fat to saturated fat ratio.^48^ In that study, participants were provided a high-fat diet (40% of total energy derived from fat) for 4 menstrual cycles, followed by a low-fat diet (20% of total energy from fat) for another 4 menstrual cycles.^48^ Altogether, evidence for the use of polyunsaturated fatty acids in PMS symptom relief is relatively weak.

#### Carbohydrates

Of the 3 studies (n = 231 participants) included in this review and involving carbohydrate-based interventions, 2 identified a positive association between carbohydrate intake and a reduction in PMS symptoms, ^42^^,^^56^ whereas the third study^64^ did not report between-group differences. Sayegh et al^56^ conducted a crossover trial comparing a carbohydrate-rich beverage, designed to increase tryptophan levels, with 2 other isocaloric beverages in a single day during the late luteal phase for 3 menstrual cycles. Results indicated a substantial decrease in PMS-related depression, confusion, and anger 90–180 minutes after consumption (*P* < 0.05).^56^ An open-label trial replaced 4 daily servings of refined grains with whole grains for 3 months. Researchers found a significant reduction in PMS-related mood and behavioral symptoms compared with the control group who continued to consume their usual diet (all *P* < 0.01).^42^ Dietary interventions promoting the consumption of higher-quality carbohydrates may improve psychological PMS symptoms, but require confirmation by additional research.

#### Other dietary interventions

In a double-blinded, crossover study investigating soy isoflavone consumption on behavioral and somatic symptoms in 23 women, researchers reported significant improvements in physical PMS symptoms (*P* < 0.05), but not psychological.^38^ Comparably, a dietitian-designed “PMS diet,” involving 111 university students, which promoted complex carbohydrates, calcium, and fish while limiting energy-dense foods, had no impact on psychological PMS symptoms after a 12-week intervention.^65^

## DISCUSSION

To our knowledge, this is the first systematic review to explore the effects of nutritional interventions from RCTs on the psychological symptoms of PMS. Consistent evidence suggests that women with PMS-related emotional symptoms may benefit from vitamin B_6_ (≥50mg/d), calcium (≥1000mg/d), and zinc (≥30mg/d) supplementation. There is limited evidence that vitamin D supplements improve PMS-associated emotional symptoms in vitamin D–replete individuals. Contradictory evidence exists regarding vitamin B_1_, replacing refined grains with whole grain carbohydrates, multivitamin supplementation, and manipulation of dietary fatty acid composition, so these interventions cannot currently be recommended for PMS psychological symptom reduction. We found that magnesium supplementation, soy isoflavones, and specially designed “PMS diets” had no effect on PMS-related psychological symptoms. However, these findings result from single studies and, therefore, cannot be considered as conclusive evidence.

Oxidative stress refers to the overproduction of reactive oxygen species, which exceeds the body’s antioxidative capacity to neutralize them.^67^ Oxidative stress has been implicated in a range of diseases, including neurodegenerative diseases, cancer, cardiovascular disease, chronic kidney disease, and, more recently, psychiatric disorders.^67^^,^^68^ Concentrations of circulating inflammatory markers, such as cytokines, increase around ovulation and peak during menstruation.^68^^,^^69^ Comprehensive research has shown that cytokines adversely impact stress response pathways and neurotransmitter function in psychological disorders that share similar characteristics with PMS.^68^^,^^69^ Consequently, investigations are underway to explore the potential roles of chronic inflammation and oxidative stress in the pathogenesis of premenstrual disorders.^69^ A cross-sectional study of 277 women revealed significant positive associations between menstrual symptom severity and levels of inflammatory markers, which were more than 2-fold greater in women with PMS than in those without.^69^ Although limited by its observational study design, the authors adjusted for potential confounding factors such as smoking, body mass index, alcohol use, and oral contraceptives.^69^ Other studies have also reported that women with PMS have lower total antioxidant capacity compared with control groups.^67^^,^^70^ Moreover, Duvan et al^70^ found increased levels of lipid hydroperoxides, a measure of oxidative stress from lipid peroxidation, suggesting a shift toward oxidant overproduction and disturbance of oxidant and antioxidant homeostasis.

Various vitamins, minerals, plant extracts, polyphenols, and omega-3 fatty acids exhibit antioxidative and anti-inflammatory properties and have been hypothesized as potential treatments for PMS and psychological disorders.^67^^,^^69^ In this review, we found that vitamin supplementation had mixed effects on the psychological symptoms of PMS. Overall, most trials investigating vitamin B_6_ supplementation reported significant reductions in negative mood and behavioral PMS symptoms.^40^^,^^41^^,^^49^ The mechanisms whereby vitamin B_6_ may alleviate PMS symptoms remain unclear. One clue lies in its role as a cofactor for neurotransmitter synthesis.^71^^,^^72^ Specifically, vitamin B_6_ can regulate the production of GABA, an inhibitory neurotransmitter that, when positively modulated, produces anxiolytic and calming effects.^71^^,^^73–75^ Furthermore, previous research indicates that increased vitamin B_6_ intake is associated with lower rates of depression and anxiety, mainly due to its positive effects on GABAergic neurotransmission, which is crucial for overall brain function and emotional well-being.^76^

We found that although vitamin D supplementation appeared to be beneficial for alleviating PMS-related emotional symptoms in women with a preexisting vitamin D deficiency, there was no evidence to support supplementation in vitamin D–replete women with PMS. In a cross-sectional study of 998 women aged 20–29 years, vitamin D deficiency was associated with greater risk of PMS-related symptoms of anxiety, mild confusion, and severe fatigue.^77^ A recent systematic review of RCTs, case-control studies, and cross-sectional studies also found that low levels of vitamin D during the luteal phase can exacerbate PMS, and supplementation may relieve adverse symptoms.^78^ More robust RCTs are needed to establish the effect of supplementation on PMS symptoms in women with sufficient levels of vitamin D.

The American College of Obstetricians and Gynecologists currently recommends calcium supplementation as a potential approach to decrease mood symptoms of PMS.^79^ We found in this review that both calcium and zinc supplementation may be beneficial dietary interventions for PMS symptom reduction. Cyclical fluctuations in extracellular calcium concentrations during the menstrual cycle are thought to be caused by ovarian steroid hormones, specifically estrogen, which has calcium-antagonistic properties.^80^ This results in reduced serum calcium levels and aberrations of neurotransmitter synthesis and release, leading to serotonin dysregulation and PMS mood symptoms.^80^ A systematic review of 8 interventional and 6 observational studies identified that women with PMS had lower levels of serum calcium and that supplementation reduced the incidence and severity of PMS.^80^ Similar to variations in circulating calcium concentrations, the decrease in serum zinc concentration during the luteal phase is further exacerbated for women with PMS, leading to mood and behavioral disorders.^81^ Zinc is proposed to relieve PMS symptoms through its antidepressant-like role, supporting serotonin activities through increased brain synthesis of BDNF, and as an antioxidant by preventing oxidative stress.^47^ Findings from a recent RCT (published in 2023 after our database search was conducted) involving 69 Iranian university students with PMS, using a 3-month intervention of 220 mg of zinc supplementation, revealed significant reductions in psychological symptoms, including depressed mood, anxiety, and anger (*P* < 0.001).^82^ Limitations of this trial included inadequate reporting of methods used to diagnose PMS and no reporting of participants’ compliance with the intervention.

PMS/PMDD mood symptoms share similarities with neuropsychiatric conditions that are associated with irregular magnesium metabolism.^83^ Thus, one hypothesis could be that insufficient magnesium levels may contribute to PMS/PMDD symptoms. However, a double-blinded, crossover RCT did not provide supportive evidence for magnesium deficiency in women with PMDD or the efficacy of intravenous (not dietary) magnesium as a treatment.^84^ Furthermore, we found in this review no significant effects of magnesium supplementation on the psychological symptoms of PMS.

Articles in this review reported contradictory effects of dietary fatty acids on the psychological symptoms of PMS; therefore, recommendations cannot be made regarding their consumption. Two long-chain omega-3 fatty acids, eicosapentaenoic acid and docosahexaenoic acid, suppress the production of pro-inflammatory cytokines and eicosanoids, which may work to alleviate psychological symptoms.^85^ A meta-analysis concluded that an omega-3 supplement with a minimum eicosapentaenoic acid to docosahexaenoic acid ratio of 60:40 (compared with 100% docosahexaenoic acid) had beneficial impacts on general depressive symptoms (unrelated to PMS),^86^ suggesting that the type and composition of omega-3 supplements may affect their efficacy.

The gut-brain axis is gaining attention for its influence on psychological disorders, like anxiety and depression, both commonly associated with PMS/PMDD.^87^ The gut microbiota plays a vital role in several physiological functions, such as maintaining optimal gut health, controlling immune system responses, production of beneficial short-chain fatty acids, and generation of neurotransmitters (ie, dopamine and serotonin).^88^^,^^89^ Takeda and Chiba^90^ explored the role of the gut microbiota in equol production from isoflavone, a phytoestrogen that induces a calming effect in the brain, potentially alleviating depression and anxiety in premenstrual disorders. In contrast, in this review, we found no significant effects of soy isoflavone on psychological PMS symptoms, although findings were limited by a single trial with high participant attrition.^38^

One trial included in this review^42^ provided evidence supporting the replacement of refined grains with whole grains for management of psychological PMS symptoms. Similarly, a cohort study of 69 954 women found that increased consumption of whole grains was associated with a significantly lower risk of depression compared with refined grains.^91^ However, a recent systematic review of 5 cross-sectional studies found no association between dietary glycemic index or glycemic load and depression.^92^ A diet rich in whole grains and fiber may exert beneficial effects on neurotransmitter metabolism, decrease chronic inflammation by mitigating lipid oxidation, and positively modulate the gut microbiota by regulating short-chain fatty acid production, potentially reducing PMS-associated mood symptoms. Additional RCTs are required to confirm the association between carbohydrate quality and the emotional symptoms of PMS.

Two recently published systematic reviews^24^^,^^93^ conducted by the same research team summarized the effect of herbal and nutritional supplements on PMS-related psychological symptoms. Both reviews largely identified the same 25 RCTs, but most of these were trials of herbal supplements. Because of the limitations inherent in the search strategy used, these reviews only identified 4 RCTs involving nonherbal nutritional supplements, making it impossible to draw any conclusions about their individual efficacy. An advantage of the present review was the use of a transparent literature search of databases from their inception, which enabled 31 RCTs of nonherbal nutritional supplements to be identified and synthesis of results of multiple studies investigating the same nutritional supplement. In contrast, Sultana et al^24^ simply pooled the results of most trials (despite each involving a unique dietary supplement) into a meta-analysis and, therefore, could only make the general conclusion that herbal and nutritional supplements may be useful for the alleviation of PMS symptoms.

### Strengths and limitations

Key strengths of this review include the analysis of high-level evidence from RCT study designs and a comprehensive search strategy. Studies were included on the basis of predefined criteria, which included the use of validated measurement tools to assess outcomes. Three independent reviewers conducted the data extraction and used the Cochrane RoB2 tool to assess the risk of bias of the included studies.

In addition to these strengths, there are also some potential areas for improvement. Although it may also be considered a strength, 1 limitation may have been the exclusion of non-RCT studies from this review. Current literature evaluating nutrition interventions for PMS symptoms are limited, and including the results of observational studies perhaps could have provided a deeper understanding of the relationship between nutritional intake and the psychological sequelae of PMS/PMDD. Because the heterogeneity between studies regarding PMS diagnostic criteria and variations in study protocols (eg, type of intervention, dose and trial duration), a meta-analysis of combined data was not possible. Some studies only reported aggregate scores for several PMS symptoms (physical, behavioral, and emotional), making the effects of nutritional intake on psychological symptoms alone impossible to determine. A number of methodological limitations of the studies included in this review were evident, and their results, therefore, should be interpreted with caution. Most studies were determined to have either some concerns of bias or a high risk of bias due to undisclosed methods of randomization or blinding, selective reporting of results, or failure to conduct intention-to-treat analyses. The Consolidated Standards of Reporting Trials Statement, which outlines clear RCT reporting guidelines, was first published in 1996^94^ and was updated in 2010.^95^ Eleven of the studies included in this review were published before 1996. This may account for the risk-of-bias concerns in several trials.

### Recommendations for future studies

The current knowledge gap in this area of women’s health highlights a clear need for more high-quality studies to understand PMS pathophysiology and the potential biological mechanisms whereby nutritional interventions may reduce adverse PMS symptoms. For this to occur, it is paramount that future studies use validated tools to diagnose PMS/PMDD, recruit an adequately powered sample size, apply intention-to-treat analyses, compare treatment groups with appropriate controls, and ensure clear reporting of methodology. It is also important that future studies are preregistered in a clinical trials registry with a statistical analysis plan.

## CONCLUSION

The present body of scientific literature investigating the effects of nutritional interventions on reducing the psychological symptoms of premenstrual disorders is limited. Consistent evidence from this review suggests women with PMS-related emotional symptoms may benefit from vitamin B_6_ (≥50mg/d), calcium (≥1000mg/d), and zinc (≥30mg/d) supplementation. Long-term consumption of vitamin B_6_ supplements in high doses (>100mg/d) has been associated with adverse side effects such as peripheral neuropathy, therefore intake for premenstrual symptom relief should be balanced with consideration of potential toxicity. There is only limited or no evidence to support the use of vitamin B_1_, vitamin D, whole-grain carbohydrates, soy isoflavones, dietary fatty acids, magnesium, multivitamin supplementation, or PMS-specific diets. However, due to the heterogeneity and risk of bias of the available evidence, more high-quality investigations are necessary to confirm whether dietary intervention or nutritional supplementation is an effective strategy to lessen the burden of menstrual-associated emotional symptoms for millions of women worldwide.

## Supplementary Material

nuae043_Supplementary_Data

## Supporting Information

The following Supporting Information is available through the online version of this article at the publisher’s website.

*Table S1* Database search strategy used in this systematic review to explore the effect of nutrition-based interventions on psychological symptoms of premenstrual syndrome in women
