# Supplementary material for: Effect of nutritional interventions on the psychological symptoms of premenstrual syndrome in women of reproductive age: a systematic review of randomized controlled trials
Source: Nutr Rev. 2024 Apr 29;83(2):280–306. doi: 10.1093/nutrit/nuae043 (PMC11723155; doi:10.1093/nutrit/nuae043)
Supplement: nuae043_Supplementary_Data [file nuae043_supplementary_data.docx]

**Table S1.** Database search strategy used in this systematic review to explore the effect of nutrition-based interventions on psychological symptoms of premenstrual syndrome in women.

| **#** | **Search term (Example for Ovid MEDLINE, search conducted 22/10/22)** |
| --- | --- |
| 1 | exp Diet/ |
| 2 | exp Food/ |
| 3 | exp Nutrients/ |
| 4 | exp Vitamins/ |
| 5 | exp Minerals/ |
| 6 | exp Dietary Supplements/ |
| 7 | exp Feeding Behavior/ |
| 8 | exp "diet, food, and nutrition"/ |
| 9 | (nutrition* adj3 (therap* or supplement* or intervention* or plan* or prescri* or educat* or program or advice or support or replacement or substitut* or pattern* or intake or habit)).tw. |
| 10 | (diet* adj3 (therap* or supplement* or intervention* or plan* or prescri* or educat* or program or advice or support or replacement or substitut* or pattern* or intake or habit)).tw. |
| 11 | (food* or vitamin* or mineral* or diet* or drink or beverage).tw. |
| 12 | exp Premenstrual Syndrome/ |
| 13 | (premenstrua* adj5 syndrome*).tw. |
| 14 | premenstrual.tw. |
| 15 | PMS.tw. |
| 16 | (premenstrua* adj5 tension*).tw. |
| 17 | PMT.tw. |
| 18 | exp Menstruation Disturbances/ |
| 19 | exp Premenstrual Dysphoric Disorder/ |
| 20 | (late luteal phase adj5 disorder).tw. |
| 21 | PMDD.tw. |
| 22 | dysphor*.tw. |
| 23 | 1 or 2 or 3 or 4 or 5 or 6 or 7 or 8 or 9 or 10 or 11 |
| 24 | 12 or 13 or 14 or 15 or 16 or 17 or 18 or 19 or 20 or 21 or 22 |
| 25 | 23 and 24 |
